# Supplementary material for: Trained Immunity Based-Vaccines as a Prophylactic Strategy in Common Variable Immunodeficiency. A Proof of Concept Study
Source: Biomedicines. 2020 Jul 9;8(7):203. doi: 10.3390/biomedicines8070203 (PMC7400202; doi:10.3390/biomedicines8070203)
Supplement: Supplementary file 1 [file biomedicines-08-00203-s001.pdf]

SUPP. TABLE 1

Subjects	Antibiotic consumption		Unscheduled outpatient visits		Work absenteeism	
	Courses before MV130	Courses after MV130	Visits before MV130	Visits after MV130	Days before MV130	Days after MV130
#1	5	1	5	2	3	1
#2	3	0	2	0	0	0
#3	5	1	5	1	2	0
#4	7	4	6	3	3	2
#5	4	0	3	0	1	0
#6	5	1	5	1	2	0
#7	4	0	5	0	0	0
#8	5	1	3	1	2	0
#9	7	1	5	0	3	2
#10	3	1	5	3	0	0

SUPP. FIGURE 1

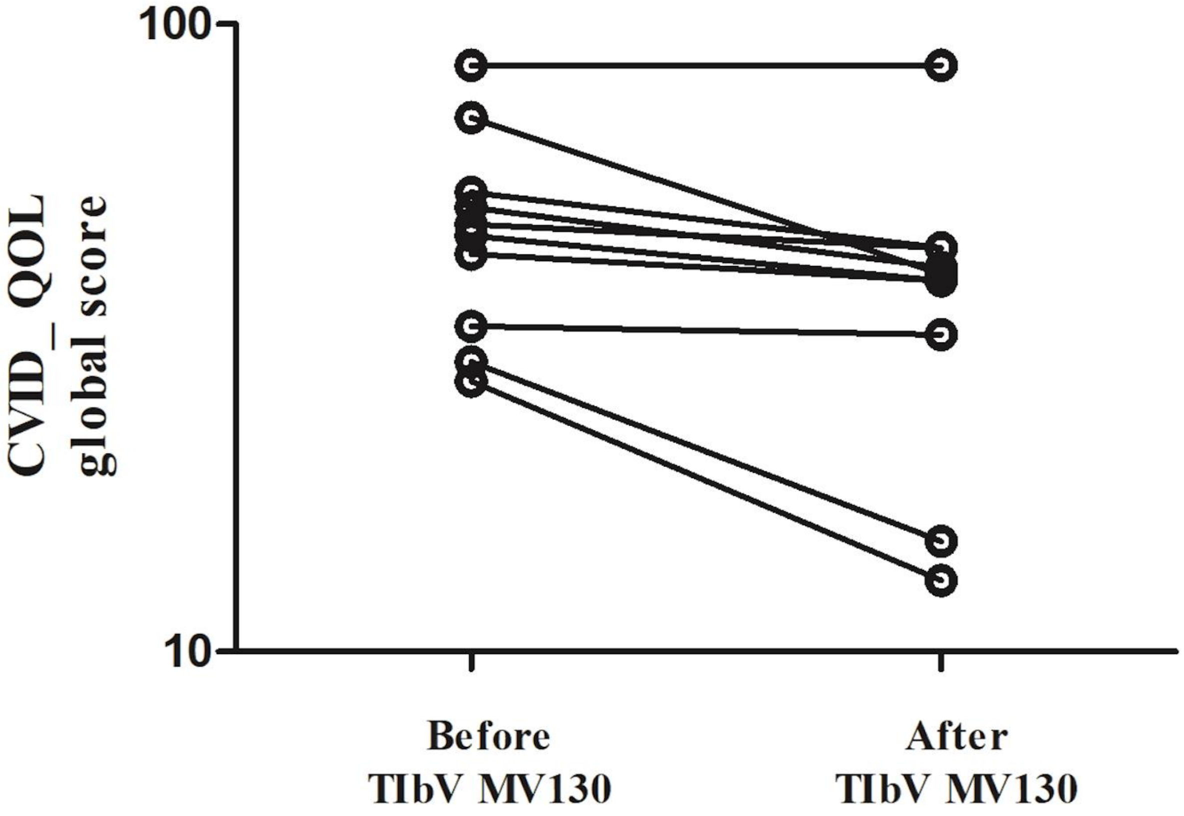

SUPP. TABLE 2

Type of cost	Costs for	Specification (Care for infectious episodes)	Source of resources used	Source of costs per 'unit'	Cost calculation
Medical direct costs	Primary care	General Practitioner and/or nurse	Self-reported in CRF and/or information recorded in EHR or medical record, if it is available	institute of Health provider	Number of visits × price
		Nurse visit	Self-reported in CRF and/or information recorded in EHR or medical record, if it is available	Institute of Health provider	Number of visits × price
		Emergency room visits	Self-reported in CRF and/or information recorded in EHR or medical record, if it is available	Institute of Health provider	Number of visits × price
		Inpatient costs	Self-reported in CRF and/or information recorded in EHR or medical record, if it is available	Institute of Health provider	Services provided × price
		Admission to acute-care nonsurgical departments	Self-reported in CRF and/or information recorded in EHR or medical record, if it is available	Institute of Health provider	Services provided × price
	Diagnosis test	Diagnostic imaging	Self-reported in CRF and/or information recorded in EHR or medical record, if it is available	Institute of Health provider	Services provided × price
		Laboratory analysis	Self-reported in CRF and/or information recorded in EHR or medical record, if it is available	Institute of Health provider	Services provided × price
	Pharmaceutical treatment	Antibiotics and other prescription medications	Self-reported in CRF and/or information recorded in EHR or medical record, if it is available	Standard Pharmaceutical Prices by Consejo General de Colegios Oficiales de Farmacéuticos	Medicines bought × price medicine
	Other costs	Medical devices (infusion pumps)	Self-reported in CRF	Patient	Number of medical device × price per medical device
Non-medical direct costs		Travel expenses	Self-reported in CRF	Patient	
		Home care	Self-reported in CRF	Patient	Hours of aid × price per aid
Indirect cost	Absenteeism	xx	Self-reported in CRF	Self-reported, profession classification assigned according to Spanish National Institute of Statistics	Days of work sick leave × salary
	Presenteeism	xx	Self-reported in CRF	Self-reported, profession classification assigned according to Spanish National Institute of Statistics	Reduction percentage × productivity
